# microRNA-206 impairs c-Myc-driven cancer in a synthetic lethal manner by directly inhibiting MAP3K13

**DOI:** 10.18632/oncotarget.7653

**Published:** 2016-02-24

**Authors:** Han Han, Yuxing Chen, Li Cheng, Edward V. Prochownik, Youjun Li

**Affiliations:** ^1^ College of Life Sciences, Medical Research Institute, Wuhan University, Wuhan 430072, China; ^2^ Division of Hematology/Oncology, Children's Hospital of Pittsburgh of UPMC and The Department of Microbiology and Molecular Genetics, The University of Pittsburgh Medical Center, Pittsburgh, Pennsylvania 15224, USA

**Keywords:** Myc, microRNA, MAP3K13, breast cancer

## Abstract

c-Myc (Myc) is one of the most frequently dysregulated oncogenic transcription factors in human cancer. By functionally screening a microRNA (miR) library, we identified miR-206 as being a synthetic lethal in Myc over-expressing human cancer cells. miR-206 inhibited MAP3K13, which resulted in Myc protein de-stabilization, and an inhibition of anchorage-independent growth and *in vivo* tumorigenesis by Myc over-expressing human cancer cells. Eliminating MAP3K13 by shRNA recapitulated the effects caused by miR-206, thus supporting the idea that miR-206's effect on Myc was mediated through MAP3K13. Conversely, enforced expression of MAP3K13 stabilized Myc by promoting its N-terminal phosphorylation and enhancing its transcriptional activity. Gene expression analyses of breast cancers expressing high levels of Myc indicated that low miR-206 expression and high MAP3K13 expression correlated with poor patient survival. The critical link between miR-206 and MAP3K13 in the development of Myc over-expressing human cancers suggests potential points of therapeutic intervention for this molecular sub-category.

## INTRODUCTION

Myc behaves as a transcriptional activator or repressor of gene expression in a wide range of cell types [1–3]. Myc expression is tightly controlled, both at the transcriptional level and post-translationally [1–3]. Myc is phosphorylated at Ser62 (pS62) in response to growth signals and this modification increases its protein stability and oncogenic activity [4–7]. Through its downstream targets, Myc promotes growth, metabolism and oncogenesis. Myc is among the most frequently altered oncogenes in human cancers, including breast cancers [1–3, 9–11]. It occurs as a consequence of chromosomal translocation, gene amplification, point mutation, protein stabilization, or activation of upstream signaling factors [1–3, 9–12]. Myc has been shown to be necessary for cancer progression and maintenance and its upregulation is significantly correlated with aggressive tumor phenotypes and poor clinical outcomes in breast cancer and other neoplasms [1–3, 9–11]. Myc's role in the pathogenesis of cancer makes it an important, albeit elusive target of cancer treatment [10, 11, 13–15]. Because Myc is essential for the growth of normal as well as transformed cells, it has sometimes been viewed as being a poor therapeutic target given that the protein is seldom mutated in cancer and possesses no obviously “druggable” domains [13–20]. Thus it has been suggested that taking advantage of Myc's synthetic lethal interactions could be exploited as an effective therapeutic strategy in Myc-driven human cancers [13–25].

microRNAs (miRs) are short non-coding RNAs that post-transcriptionally inhibit the translation and/or stability of mRNAs [26]. miRs can function as oncogenes or tumor suppressors [27–29]. It has been reported that antagomirs and the re-expression of tumour-suppressive miRs can inhibit tumor growth, thus suggesting that manipulating miR levels is a viable therapeutic approach in cancer with limited side effects *in vivo* [27–29].

We report here the results of a high-content screen to identify miRs that selectively inhibit the growth of Myc over-expressing human cancer cells. miR-206 was identified as one such miR that exerted a potent anti-proliferative effect in a model *in vitro* system. Further investigation has revealed that the link between miR-206 and Myc involves the suppression of MAP3K13, a member of the mitogen-activated protein kinase kinase kinase (MAPKKK) family that has previously been reported to activate both the NF-κB and JUN pathways [30]. By inhibiting MAP3K13, miR-206 inhibits the stability of Myc protein and thus of tumor cell survival and proliferation.

## RESULTS

### Functional screening identifies miR-206 as a specific inhibitor of Myc-over-expressing cells

**Figure 1:**
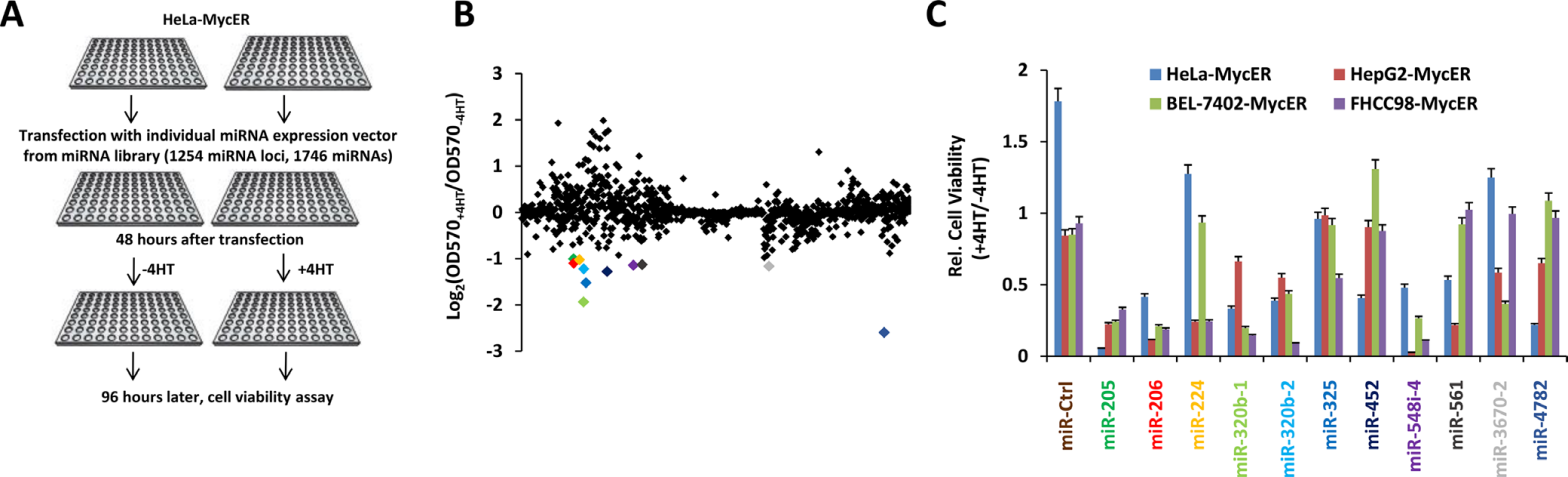
Functional screening identifies miRs that specifically inhibit the growth of Myc overexpressing cells (**A**) Screening procedure. HeLa cells stably expressing MycER were transfected with individual miR expression vectors from the miR library, cultured in the absence or presence of 4-OHT to activate MycER and cell viability was determined. (**B**) Results of the high-content functional library screening for miRs specifically inhibiting growth of Myc overexpressing cells. miR-206 is indicated in red. (**C**) The effects of selective miRs inhibiting growth of several Myc overexpressing cells. The experiments were performed following the protocol described in (A). Data are means ± SD.

To identify miRs that inhibit the growth of Myc over-expressing cells, we performed an initial high-content, function-based miR screen in HeLa cells stably transfected with an inducible Myc-estrogen receptor fusion gene (MycER). The miR library used was comprised of 1254 individual miR expression vectors generated by our lab. We used an MTS assay to identify those miRs that inhibited cell growth following MycER activation mediated by 4-hydroxytamoxifen (4-OHT). In an initial screening, 11 miRs showed marked inhibitory effects on the growth of this cell line (Log_2_ relative growth ratio < −1, Figure 1A–1B and Supplementary Table 1). After confirming the growth inhibitory effects of these miRs in HeLa-MycER cells, we tested them in three additional MycER-cell lines, HepG2, BEL7402 and FHCC98. Three miRs, miR-205, miR-206 and miR-548i-4 significantly inhibited all four cell lines by greater than two-fold under conditions of MycER activation (Figure 1C).

**Figure 2:**
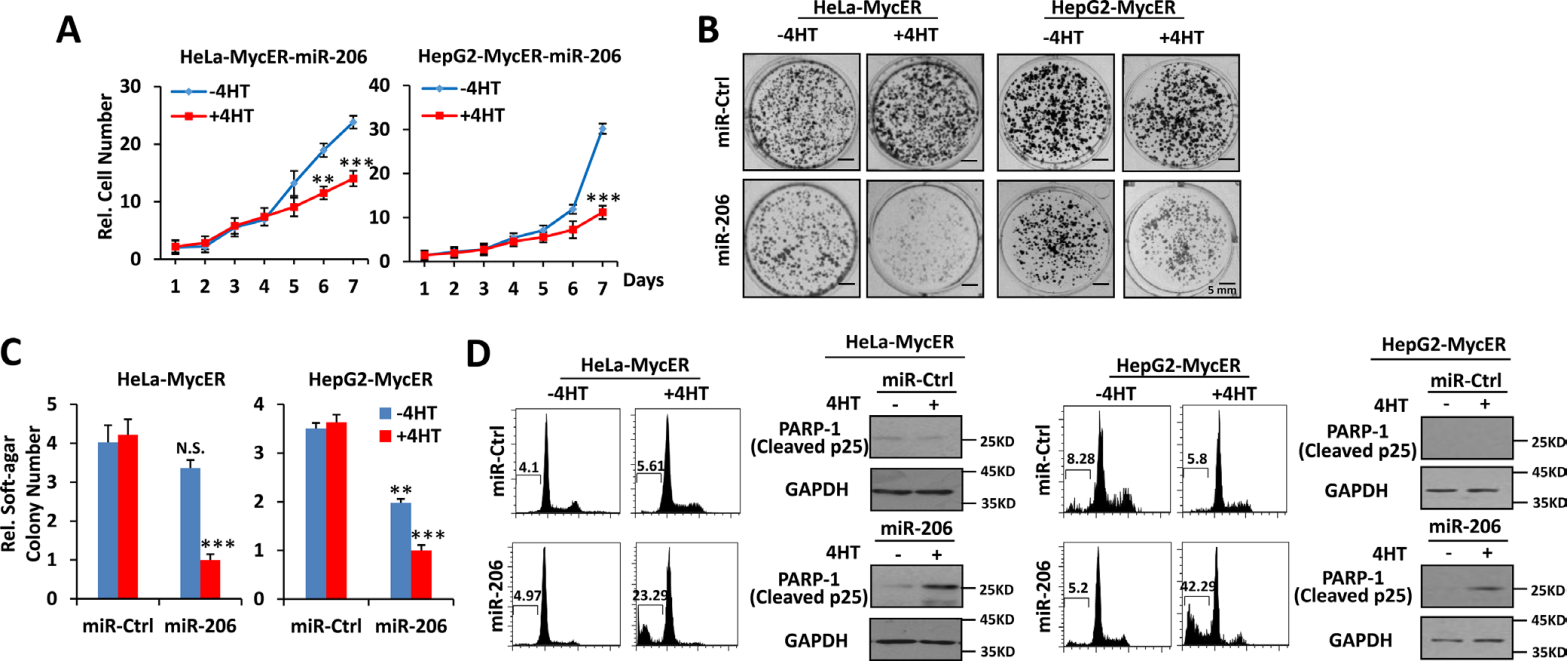
Overexpression of miR-206 is synthetically lethal with Myc overexpression (**A**–**C**) Over-expression of miR-206 inhibits growth, focus formation and anchorage-independent soft agar growth of Myc over-expressing cells. (**D**) Over-expression of miR-206 promotes apoptosis of Myc overexpressing cells. Hela-MycER and HepG2-MycER cells stably transfected with control or miR-206 expression vectors were used to construct growth curves to assess focus formation and anchorage-independentsoft agar growth. Apoptosis was quantified by calculating the SubG1 DNA content and PARP-1 expression. Results are presented as means ± SD. *P < 0.05, **P < 0.01, ***P < 0.001.

To study further the effects of miR-205, miR-206 and miR-548i-4 on Myc over-expressing cells, Hela-MycER and HepG2-MycER cells were stably transfected with expression vectors for each miR and the effects of MycER induction on cell growth, anchorage-independent growth and survival were measured. In the presence of MycER activation, the ectopic expression of miR-206 significantly inhibited both anchorage-dependent and -independent growth (Figure 2A–2C) and promoted robust apoptosis (Figure 2D) relative to a control miR. Although miR-206 did inhibit focus formation and anchorage independent soft agar growth somewhat in control HeLa-MycER and HepG2-MycER cells in the absence of 4-OHT, this effect was much greater in the presence of the compound (Figure 2B and 2C). Ectopic expression of miR-205 also displayed similar effects (Supplementary Figure 1A–1D). In contrast, the ectopic expression of miR-548i-4 failed to show any effects on the growth of HeLa-MycER cells (Supplementary Figure 1A). Thus, of the three miRs identified in our initial HeLa-MycER screen, miR-205 and miR-206 demonstrated the most wide-ranging and robust synthetic lethal effect on Myc over-expressing cancer cells.

### miR-206 inhibits tumor growth of human cancer cells with high Myc levels

**Figure 3:**
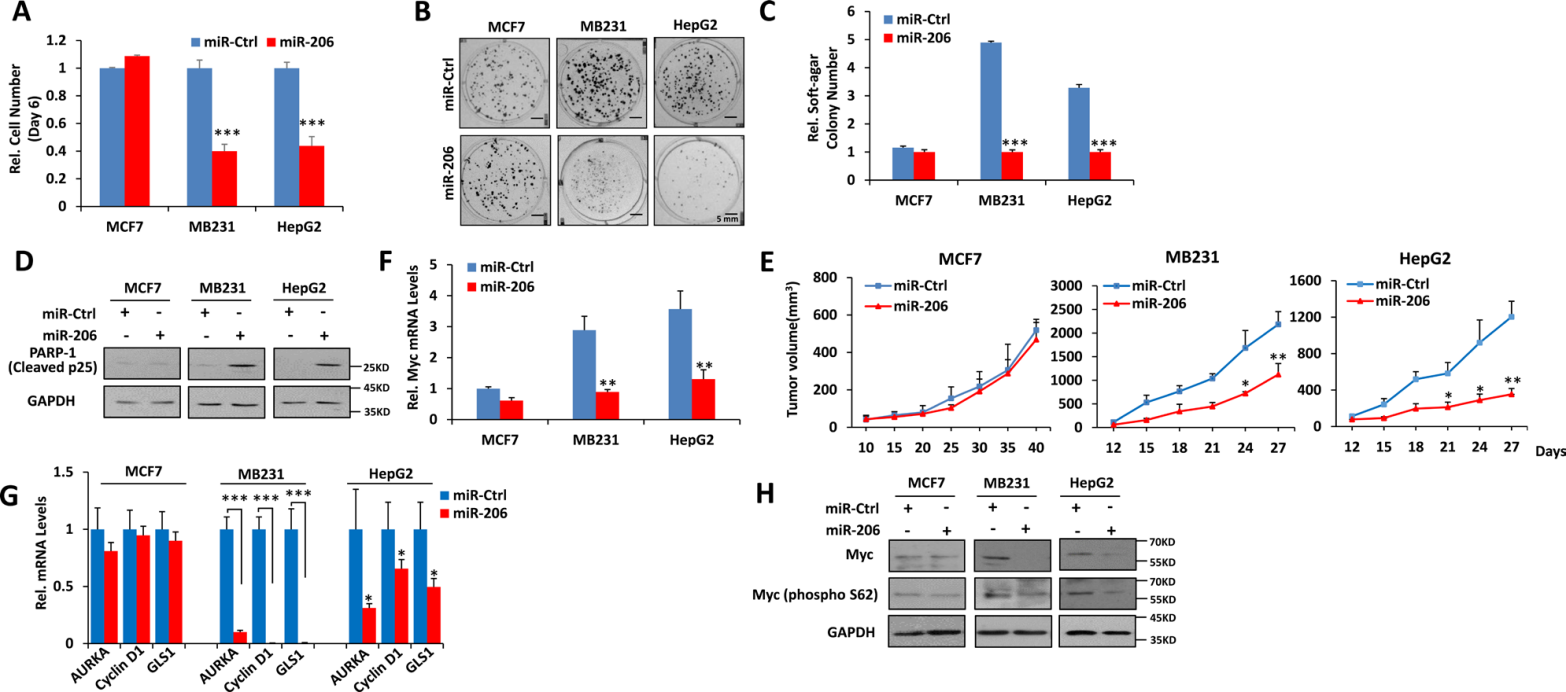
miR-206 impairs growth, clonogenicity and tumorigenicity of cancer cells expressing high levels of Myc (**A–C**) miR-206 inhibits growth, focus formation and anchorage-independent soft agar growth of Myc-over-expressing human cancer cells. (**D**) Over-expression of miR-206 promotes apoptosis of Myc-over-expressing human cancer cells. MCF-7, MB231 and HepG2 cancer cells stably expressing miR-206 or a control miR were used as described above for growth curves, focus formation, anchorage-independent soft agar growth and apoptosis detection. (**E**) Over-expression of miR-206 impairs tumorigenicity of Myc-over-expressing human cancer cells. The above cells were inoculated into nude mice. Tumor growth was monitered over time. (**F**) miR-206 decreases Myc mRNA levels of Myc-over-expressing human cancer cells. (**G**) miR-206 decreases Myc transcription activity of Myc-over-expressing human cancer cells. mRNA levels for the indicated Myc target genes were detected by qRT-PCR. (**H**) miR-206 affects stabilization of Myc protein of Myc-over-expressing human cancer cells. Cell extracts above cells were assayed by immuno-blotting using antibodies against the indicated proteins. Results are means ± SD. *P < 0.05, **P < 0.01, ***P < 0.001.

Our own studies and those of others have reported that human cancer cells are generally dependent on Myc for their proliferation and survival but express highly variable levels of the oncoprotein [20, 25, 31]. To investigate the potential roles of miR-205, miR-206 and miR-548i-4 on the growth of cell lines with different levels of endogenous Myc expression, we next stably expressed each of the three miRs in MCF-7 and MB231 breast cancer and HepG2 hepatocarcinoma cells (Supplementary Figure 2A). MiR-205 significantly decreased both anchorage-dependent and -independent growth in all three cell lines whereas miR-206 and miR-548i-4 inhibited growth only in the two cell lines with high level of Myc expression, namely MB231 and HepG2 (Figure 3A–3C and Supplementary Figure 2B–2D). From these studies and those described in Figure 2, we conclude that miR-206 exerts a more potent synthetic lethal effects than miR-205 and miR-548i-4. Therefore, we selected miR-206 for further characterization. Supporting experiments revealed that forced expression of miR-206 produced higher levels of cleaved p25, indicating induction of apoptosis in both MB231 and HepG2 cells (Figure 3D). Moreover, each of these cell lines produced slower growing tumors following subcutaneous injection into immuno-compromised mice (Figure 3E and Supplementary Figure 3A–3B). Mechanistic studies revealed that enforced expression of miR-206 significantly decreased Myc transcripts, total Myc protein and its transcription activity (Figure 3F–3H). The latter correlated with a reduction of Myc phosphorylation at Ser62 (pS62) that normally serves to stabilize the protein by prolonging its half-life (Figure 3H) [4–7]. Taken together, these data suggest that miR-206 selectively inhibits the growth of tumors expressing only the highest levels of Myc by reducing Myc transcript and protein, with the latter likely being due to protein de-stabilizination mediated by a loss of Ser62 phosphorylation.

### MAP3K13 is a direct target of miR-206

**Figure 4:**
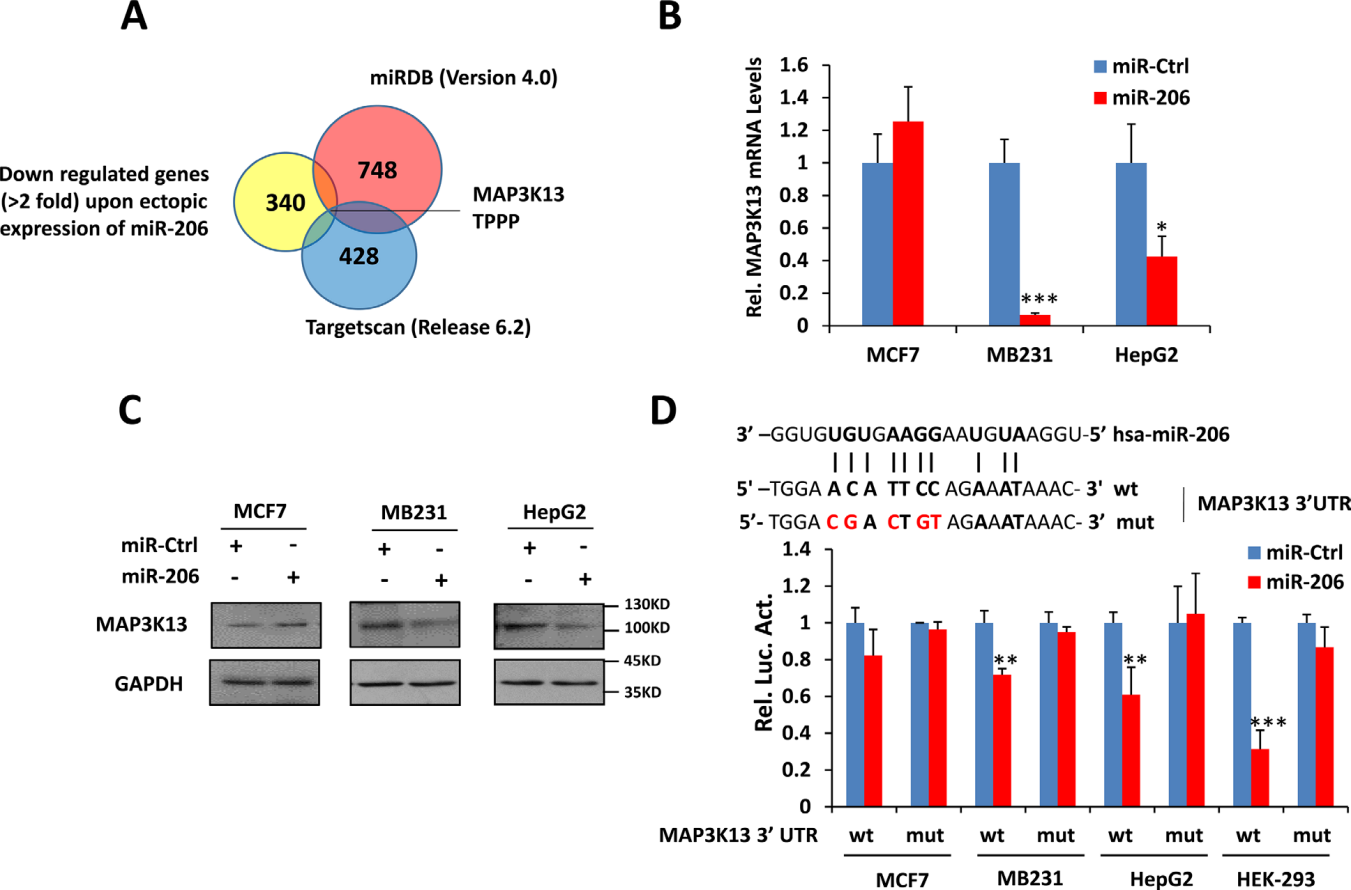
miR-206 directly targets MAP3K13 (**A**) Venn diagram showing the overlap between genes down-regulated by ectopic expression of miR-206 and genes potentially targeted by miR-206 as predicted by Targetscan (Release 6.2) and miRDB (version 4.0). (**B**–**C**) miR-206 decreases MAP3K13 mRNA and protein levels of Myc-over-expressing human cancer cells. (**D**)Upper: sequence of hsa-miR-206 and the potential miR-206-binding site at MAP3K13 3′UTR. Nucleotides mutated in the miR-206-binding site are shown in red. Bottom: luciferase assays demonstrating that inclusion of the MAP3K13 3′UTR (wt) but not a mutant version of the sequence (mut) is down-regulated by miR-206 only in cell lines over-expressing Myc. In no cases did a control miR exert any effect. Data are means ± SD. *P < 0.05, **P < 0.01, ***P < 0.001.

Identifying miR-206 targets is critical for understanding its biological functions. Therefore we integrated mRNA expression profiling with bioinformatic prediction analyses. Using mRNA microarray technology, we generated a list of down-regulated genes (2-fold threshold) in miR-206–overexpressing HepG2 cells. Further analysis of the microarray data indicated that miR-206 over-expression caused a significant preferential down-regulation of numerous predicted miR-206 targets (Supplementary Table 2). Combining this list with the candidate list produced by 2 prediction algorithms, including miRDB (Version 4.0) and Targetscan (Release 6.2) [32–33], we narrowed the miR-206 target candidates down to only 2 genes, including MAP3K13 and TPPP (Figure 4A). MAP3K13 is a member of the MAPKKK dual leucine zipper-bearing kinase family [30, 34, 35]. As expected, ectopic expression of miR-206 decreased endogenous MAP3K13 at the mRNA and protein levels in high Myc-expressing MB231 and HepG2 cancer cells but not in low Myc-expressing MCF-7 (Figure 4B–4C). We found that the 3′-UTR of MAP3K13 contains one miR-206 binding site across all database analyses (Figure 4D).

To further explore whether MAP3K13 represents a direct target of miR-206, we conducted luciferase reporter assays to determine whether the putative binding site of miR-206 in the 3′UTR of MAP3K13 is important for miR-206 mediated suppression. Indeed, ectopic miR-206 expression repressed the activity of a luciferase reporter containing a human MAP3K13 3′UTR whereas the same reporter containing a mutant miR-206 binding site was unaffected (Figure 4D). Thus, the human MAP3K13 mRNA is directly regulated by miR-206 via seed-matching sequences.

### MAP3K13 inhibition leads to tumor regression of human tumors with high Myc levels

**Figure 5:**
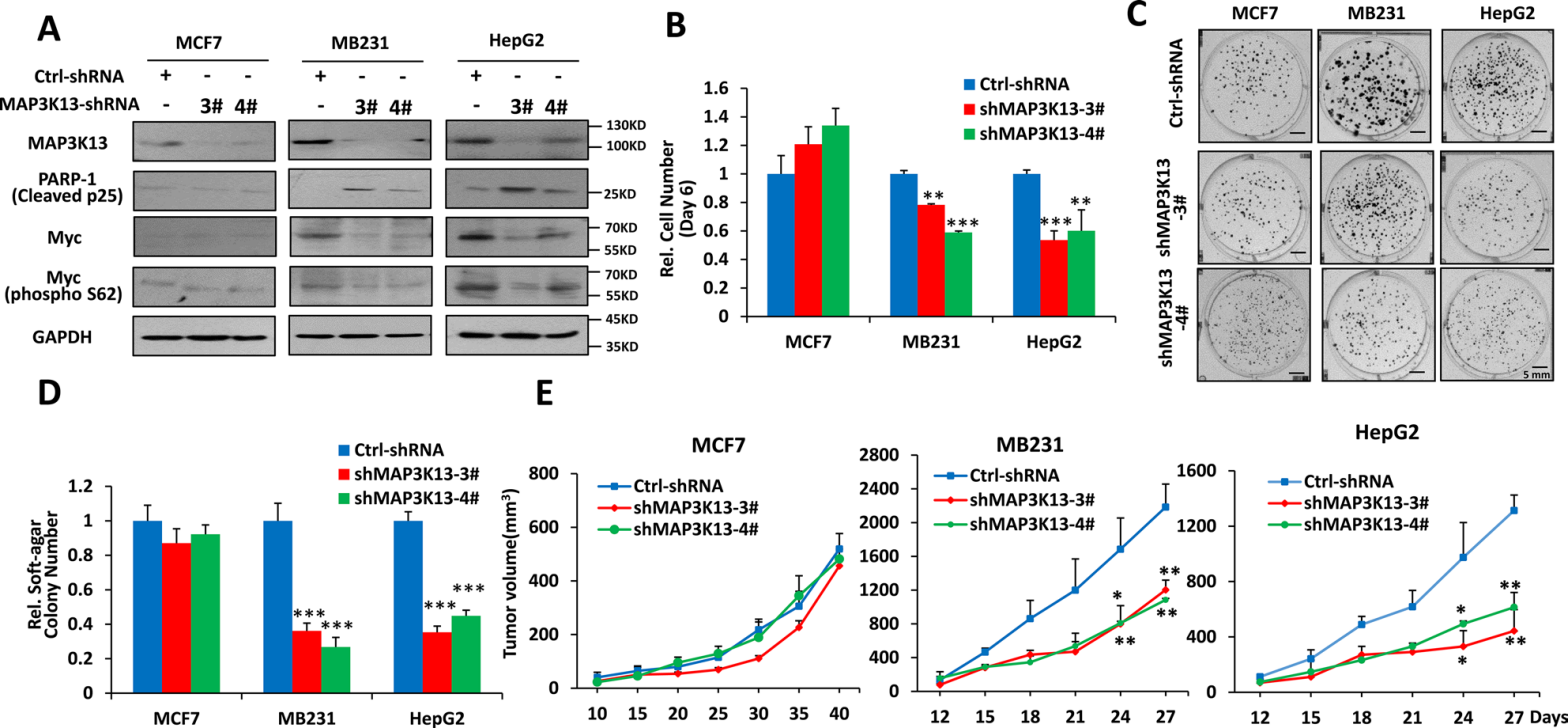
MAP3K13 is necessary for growth, clonogenicity and tumorigenicity of Myc-over-expressing human cancer cells (**A**) Depletion of MAP3K13 decreases Myc protein levels and promotes apoptosis of Myc-over-expressing human cancer cells. (**B**–**D**) Depletion of MAP3K13 inhibits growth, focus formation and anchorage-independent soft agar growth of Myc-over-expressing human cancer cells. MCF-7 and MB231 breast cancer cells and HepG2 hepatocellular carcinoma cells stably expressing MAP3K13-shRNA or control-shRNA were used to determine growth curves, focus formation, anchorage-independent soft agar growth and apoptosis. (**E**) Depletion of MAP3K13 impairs tumorigenicity of Myc-over-expressing human cancer cells. The above cells from B-D were inoculated into nude mice. Tumor growth was monitored over time.

Given that MAP3K13 appeared to be an important miR-206 target, we hypothesized that MAP3K13 would be important for Myc-driven human cancers. To evaluate the necessity of MAP3K13 for anchorage-dependent and -independent proliferation *in vitro* and tumorigenesis *in vivo*, we examined the effect of targeting MAP3K13 in MB231, HepG2 and MCF-7 cancer cells using specific shRNA. As shown in Figure 5A and Supplementary Figure 4A, targeting MAP3K13 significantly decreased protein levels in all three cancer cells but significantly inhibited growth, soft agar colony formation and *in vivo* tumorigenesis only in the high Myc-expressing lines MB231 and HepG2 (Figure 5B–5E and Supplementary Figure 4B–4C). In these two cell lines, MAP3K13 depletion also led to higher levels of cleaved p25 compared to the control group (Figure 5A). It is also important to note that the targeting of MAP3K13 in MCF7 cells did lead to a reduction of this protein, it did not lead to loss of Myc expression as it did in the other two cell lines. These results strongly supported the idea that MAP3K13 is essential for tumor growth of human cancer cells with high Myc levels and that it is the major, if not the sole, functional target of miR-206.

### MAP3K13 affects Myc protein stability

**Figure 6:**
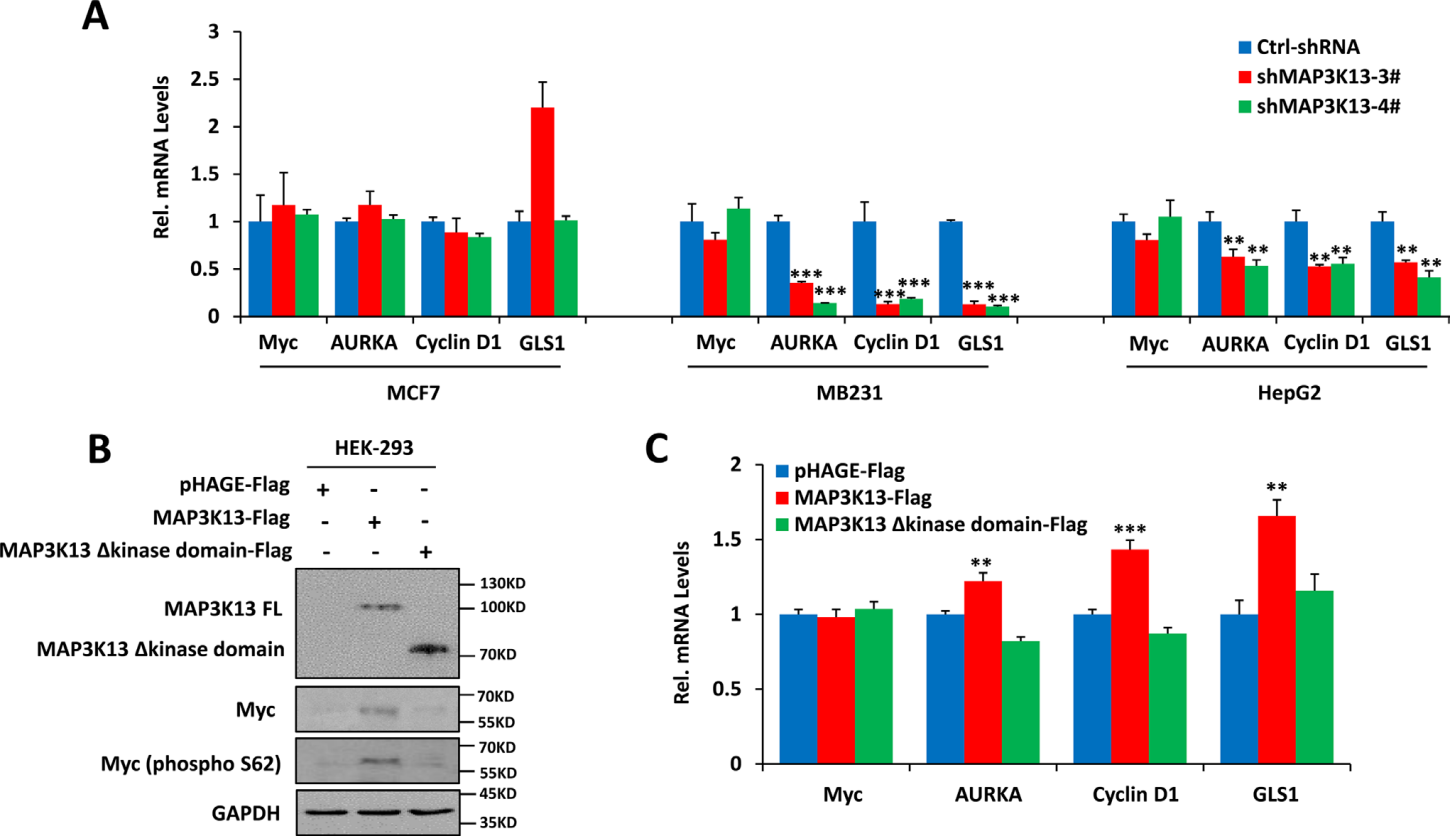
MAP3K13 affects Myc protein stability (**A**) Depletion of MAP3K13 decreases Myc target gene transcript levels. The indicated Myc target genes and Myc mRNA level were quantified by qRT-PCR. (**B**) Ectopic expression of MAP3K13 increases phospho-Myc (S62) levels and stabilizes Myc protein. Extracts from HEK293 cells transfected with wild-type or kinase mutant MAP3K13 or a control expression vector were assayed by immuno-blotting. (**C**) Ectopic expression of MAP3K13 increases Myc transcription activity. Total RNA was isolated from HEK293 cells transfected with wild-type or kinase mutant MAP3K13 or a control expression vector. The indicated Myc target gene transcripts were quantified by qRT-PCR. Results are means ± SD. *P < 0.05, **P < 0.01, ***P < 0.001.

Mechanistically, the depletion of MAP3K13, mediated by shRNA targeting of MAP3K13, decreased Myc protein and its transcription activity (Figure 5A and Figure 6A). However, depletion of MAP3K13 caused no significant effects on Myc at the protein level in MCF-7 cells with low Myc levels (Figure 5A). Further studies revealed that depletion of MAP3K13 significantly decreased the phosphorylated-S62 form of Myc leading to its protein instability and inhibiting its transcriptional activity in MB231 and HepG2 cells with high Myc levels but had no significant effects on MCF-7 cancer cells with low Myc levels (Figure 5A and Figure 6A). Furthermore, MAP3K13 expression increased the level of Ser62 Myc, stabilized the protein and improved its transcriptional activity while enforced expression of kinase domain deleted MAP3K13 had no significant effects on the above features of Myc (Figure 6B–6C). Taken together, these data suggest that MAP3K13 regulates the level of Myc S62 phosphorylation and helps to stabilize the protein, particularly in cells expressing the highest Myc levels.

### MAP3K13 correlates with poor patient survival in Myc-high breast tumors

**Figure 7:**
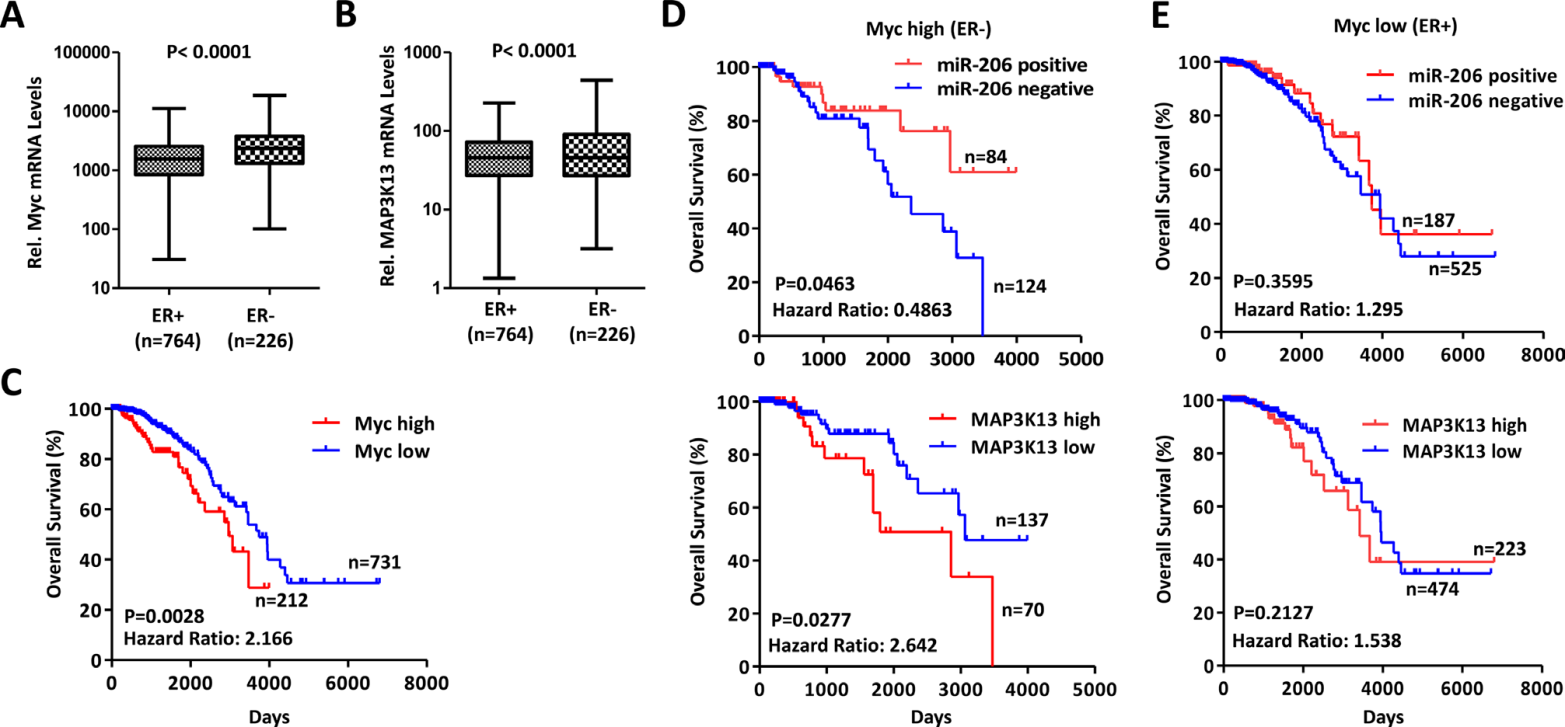
MAP3K13 is required for tumor growth of Myc-over-expressing human breast cancers (**A**–**B**) Myc and MAP3K13 are expressed at higher levels in ER negative human breast cancers. Myc and MAP3K13 mRNA expression levels were evaluated in human breast cancers from the TCGA database. Significance was performed using Mann-Whitney *U* test. The horizontal lines in the box plots represent the median, the boxes represent the interquartile range and the whiskers represent the minutes and max values. (**C**) High Myc mRNA expression correlates with poor patient survival in human breast cancers. Kaplan-Meier curves for Myc expression in association with survival from cancer patients with ER^+^ and ER^−^ tumors. (**D**) Negative miR-206 and high MAP3K13 expression correlate with poor patient survival only in ER^**-**^, Myc-high breast cancers. The expression of miR-206 is positively correlated with patient survival whereas the expression of MAP3K13 is inversely correlated. (**E**) miR-206 and MAP3K13 expression doesn't correlate with patient overall survival selectively in ER^**+**^, Myc-low breast cancers. (C–E) Significance was performed using Log-rank test.

A strong correlation has been reported between elevated Myc expression and triple-negative breast cancer [10, 11, 36, 37]; although there is also considerable heterogeneity with regard to Myc activation and receptor status [36, 37]. We thus determined independently whether Myc levels correlated inversely with survival in breast cancer and also examined the relationship of Myc levels to those for MAP3K13. To test the above hypothesis, we extracted breast cancer data sets (*n* = 1044 patients) from the Cancer Genome Atlas (TCGA) data portal for which there was accessible gene expression information [38–40]. Myc and MAP3K13 mRNA expression were generally higher in patients with ER- compared to with ER+ group (Figure 7A–7B). Patients with ER- and ER+ were then assigned as having high (*n* = 226) or low Myc expression (*n* = 764), respectively. Within these groups, patients with high expression of Myc had inferior overall survival (Figure 7C).

Our data indicate that miR-206 inhibits Myc-driven human cancer by directly targeting MAP3K13. If so, these data would predict that Myc-high human breast cancers with correspondingly high MAP3K13 or low miR-206 expression may correlate with poor survival. To examine this more carefully, we assigned patients to two groups based upon their levels of MAP3K13 expression. The first group (approximately 1/3 of patients) were classified into a high MAP3K13 gene expression group whereas the remaining patients were assigned to the low expression group. For miR-206, patients within the detected signal and non-detected signal were then assigned as having positive or negative gene expression, respectively. We then determined whether levels of miR-206 or MAP3K13 correlated with patient survival in the Myc-high or Myc-low groups. In patients with Myc-high tumors, those with negative miR-206 expression and high expression of MAP3K13 had significantly worse survival than those with positive miR-206 and low MAP3K13 expression, respectively. However, these parameters did not hold for patients with Myc-low tumors (Figure 7D–7E). These data indicate that Myc activation leads to a decreased and increased dependency on miR-206 and MAP3K13 in human breast cancers, respectively.

## DISCUSSION

Because miRs are very stable, easy to synthetize and can be readily introduced into cells, they have been viewed as having potential for cancer therapy [26–29]. miR-206 was first reported to exert significant growth inhibition and to promote cell death in human breast, lung and liver cancers [41–43]. We extended this by demonstrating that miR-206 has a similar growth inhibitory effect on both the *in vitro* and *in vivo* proliferation of breast cancer cells, but only when they express high levels of Myc as would be expected of a classic synthetic lethal relationship (Figure 3) [21–25]. This appears to be dependent on miR-206's ability to target MAP3K13 as indicated by our ability to recapitulate similar phenotypes by suppressing MAPK3K13 directly using an shRNA-based approach. miR-206 directly decreases MAP3K13 mRNA and protein levels. MAP3K13 increases pS62 phosphorylation leading to Myc stabilization and accumulation. Thus, while miR-206 likely has multiple additional targets other than MAP3K13, it is this one in particular that appears to be the most important for generating a synthetic lethal effect in conjunction with Myc over-expression (Figure 2B and 2C). These findings are consistent with our bio-informatics-based analyses of gene expression profiles from the TCGA collection of breast cancers indicating that those tumors with the highest Myc levels tend, as a group, to express the lowest levels of miR-206 and the highest levels of MAP3K13 while simultaneously being associated with more adverse clinical outcomes. miR-206 also decreases Myc mRNA level (Figure 3F) and it is possible miR-206 through other pathway to affect Myc in mRNA level. But at least we found that miR-206's effect on Myc was also mediated through MAP3K13.

Myc is an important regulator of growth in breast cancers, particularly those of the so-called “triple negative” class [1–3, 9–11]. Moreover, in some murine models of Myc-dependent breast cancer, the suppression of Myc leads to tumor regression, thus indicating that continuous Myc expression is necessary to maintain tumor growth and/or viability [44]. For all the above reasons, Myc is an attractive target for cancer therapy [13–15]. Strategies aimed at inhibiting either Myc itself, or components with which Myc associates, have been investigated as novel options for treating Myc-driven cancers as have other synthetic lethal approaches [13–15, 21–25]. Our own results are consistent with previous RNAi screens for genes with a synthetic lethal relationship to Myc. These have included regulators of the Myc network, components of transcription initiation and elongation, proteins involved in kinases and protein modification with roles in DNA repair and cell cycle checkpoints [13–15, 21–25, 45–47]. For example, the targeting of SAE2, which plays an important role in DNA repair and is necessary for the growth of cancers with high Myc levels, has been shown to interact with de-regulated Myc in a synthetic lethal manner [25].

Driver mutations of MAP3K13 have been identified previously in breast cancer, whereas inactivating mutations may abrogate signaling pathways that increase JUN kinase function in breast cancer [30, 34, 35]. Our findings show that depleting MAP3K13 significantly decreased the amount of phosphorylation on Ser62 of Myc, resulting in increased protein instability and an associated loss of transcriptional activity in cancer cells with the highest levels of Myc. Our findings also implicate MAP3K13 as being essential for tumor growth by these cells and that its levels correlate with poor patient survival in Myc-high human breast tumors. All these data strongly support the idea that miR-206 and MAP3K13 inhibits and promotes development of breast cancer by ultimately controlling Myc protein level, respectively. The synthetic lethal relationship between Myc and the miR-206-MAP3K13 pathway likely reflects a different gene expression profile that exists in tumors with the highest levels of Myc expression. A subset of these changes would appear to be responsible for imparting a greater reliance on MAP3K13 such that any reductions in its level leads to apoptotic demise. However, it is important to concede that factors other than those involving Myc protein stability may also explain its synthetic lethal relationship with MAP3K13.

In summary, our study has demonstrated a significant role for miR-206 and MAP3K13 in regulating the viability of breast cancer cells with high levels of Myc expression. Future studies should focus on the phenotypes of MAP3K13 transgenic and knockout mice and whether miR-206 suppresses Myc-mediated tumorigenesis only in breast cancer or is a more general regulator.

## MATERIALS AND METHODS

### Reagents and plasmids

4-Hydroxytamoxifen (4-OHT, H6278) was purchased from Sigma-Aldrich. All other analytical grade reagents were obtained from commercial sources. pBabe-MycER plasmid was obtained from Addgene (Cambridge, MA) (Addgene plasmid 19128). Human Pri-miR-206, including approximately 400 bp of stem-loop structures, was polymerase chain reaction (PCR)-amplified from genomic DNA and cloned into the lentiviral vector pHAGE-CMV-MCS-PGK-GFP. The human MAP3K13 coding sequence was amplified and cloned into the lentiviral vector pHAGE-CMV-MCS-PGK-puro. MAP3K13 shRNA oligonucleotides for short hairpin RNAs were designed following the principles previously described, synthesized by GENECHEM (Shanghai, China), annealed and then inserted into hU6-MCS-Ubiquitin-EGFP-IRES-puromycin (GV248). The Non-Specific sequence 5′-TTCTCCGAACGTGTCACGT-3′ was used as control. The plasmid sequence was verified by direct sequencing. The sequences of all primers including real-time PCR primers are provided in Supplementary Table 3.

### Construction of miR expression library

Human miR expression vectors were performed according to the protocols recommended by the manufacturer (BLOCK-iT Pol II miR RNAi Expression Vector Kits, Invitrogen). Human pri-miR loci, including approximately 400 bp containing stem-loop structures, were PCR-amplified from genomic DNA and cloned into BamHI and XhoI or BglII and SalI sites of pcDNA6.2-GW/EmGFP-miR vector (Catalog no. K4936-00, Invitrogen). Pri-miR loci were obtained from miRBase and primers were designed using Primer-BLAST and Primer3 [1254 miR genomic loci, miRBase release 18.0 (2012), http://mirbase.org]. PCR was performed using Pfu polymerase (Clontech) [31, 48].

### Cell culture and transfection

Human hepatocellular carcinoma cell lines (HepG2, BEL-7402 and FHCC98) were cultured under standard conditions in Dulbecco-modified Eagle's minimum essential medium (D-MEM) (GIBCO, Life Technologies, Carlsbad, CA) supplemented with 10% fetal bovine serum (FBS, GIBCO), 1% l-glutamine, 1% penicillin-streptomycin and 1% nonessential amino acids in a 5% CO2-humidified chamber at 37°C. HEK293, human breast cancer cells (MCF7 and MDA-MB-231) and cervical cancer HeLa cells were cultured as described for HCC cells [31, 48].

For transfection, the cells were seeded 1–2 days ahead of time and grown to 70–80% confluence by the time of transfection. The dosage of siRNA, shRNA or expression vectors used was determined according to the manufacturer's protocol. For transfection in six well plates, siRNA or shRNA (100 nM, final concentration), or plasmid (3–5 μg) in 50 μl Opti-MEM (GIBCO) was mixed with 5 μl of transfection reagent (Lipofectamine, Invitrogen). After allowing complexes to form for at least 20 min, they were added to 900 μl of Opti-MEM, which was then added to the cells for 6 hr before being replaced with the original cell culture medium.

Stable cell lines expressing the indicated miRs, cDNAs or shRNAs were generated by retroviral or lentiviral transduction in the presence of 8 μg/mL polybrene followed by selection with blasticidin, puromycin, or G418 for at least 10 days or were sorted by FACS. Stable cell lines were examined for the expression of miR, mRNA or shRNA expression by real-time PCR or Western blot.

### miR screen

HeLa, HepG2, BEL-7402 and FHCC98 cells stably expressing Myc-ER were seeded in triplicate at a density of 10^4^ cells per well in 96-well plates and transfected with 200 ng of individual miR expression vectors using a standard Lipofectamine 2000 transfection protocol. After 48 hours, 300 nM of 4-OHT was added. Cell viability was measured 96 hours later using the CellTiter MTS assay according to the manufacturer's instructions (Promega) and compared to parallel wells which did not received 4-OHT.

### RNA isolation, RNA quantification and cDNA microarray

RNA was isolated from the indicated cell lines using TRIzol (Invitrogen, Carlsbad, CA) according to protocols recommended by the manufacturer. DNA contamination was removed with RNAse-free DNase I. Stem-loop RT for miRs was performed according to the protocols recommended by the manufacturer. For RT-PCR, total RNA was transcribed into cDNA using random primers and reverse transcriptase (Promega). All reagents for stem-loop RT were obtained from Promega Inc. (Madison, WI) and RiboBio Co., Ltd. U6 RNA was used as an internal control. Quantitative RT-PCR was performed with SYBR Green (Bio-Rad, Hercules, CA). Primers and other reagents of miR assays were purchased from RiboBio Co., Ltd. Primers for qRT-PCR for other genes are listed in Supplementary Table 3 and GAPDH was used as an internal control.

cDNA microarray experiments were performed at the KangChen Bio-tech (Shanghai, China), using Agilent Human 4×44K Gene Expression Microarrays v2, including 27,958 Entrez Gene RNAs [48]. Briefly, total RNA from each sample was amplified and transcribed into fluorescent cRNA with using the manufacturer's Agilent's Quick Amp Labeling protocol (version 5.7, Agilent Technologies). The labeled cRNAs were hybridized onto the Whole Human Genome Oligo Microarray (4×44K, Agilent Technologies). After washing, the arrays were scanned with an Agilent Scanner G2505C. Agilent Feature Extraction software (version 11.0.1.1) was used to analyze acquired array images and to normalize the raw 2-color data in a lowess method. Subsequent data processing was performed using GeneSpring GX v11.5.1 software package (Agilent Technologies).

### Prediction of miR targets and GenBank accession numbers

The GenBank accession numbers used for c-Myc, MAP3K13 and miR-206 were NM_001706, NM_004721 and NR_029713, respectively. Computational prediction of miR targets was performed in online databases miRDB (http://www.miRdb.org/, Version 4.0) and targetscan (http://www.targetscan.org/, Release 6.2). MiRBase (http://www.miRbase.org/) was used to analyze miR information [31, 48].

### Luciferase reporter assays

Luciferase reporter assays were performed as previously described [41, 42]. The MAP3K13 3′ UTR was PCR amplified from oligo-dT–primed cDNA of HEK293 cells, inserted into pGL3-basic vector under control of the HSV-TK promoter, and verified by sequencing. Mutations in the miR-206 seed-matching sequences were generated by overlap extension PCR. Primers for the constructs and mutations are listed in Supplementary Table 3. For luciferase assays, MCF7, MB-231, HepG2 and HEK293 cells were seeded in 24-well format dishes and transfected with the indicated firefly luciferase reporter plasmid, Renilla reporter plasmid, in a PGL3-vector with HSV TK Promoter, as a normalization control and miR-206 or a negative control expression vector. Luciferase activity was measured after 2 days and analyzed using the Dual Luciferase Reporter assay (Promega).

### Colony formation, soft agar growth, and cell cycle and subG1 DNA content

For colony formation assays, 10^3^ cells were seeded in triplicate into 6-well plates and grown for two weeks. Colonies were counted and photographed after methanol fixation and methylene blue staining. For soft agar colony assays, 4 × 10^3^ cells were plated in triplicate in soft agar (0.35% low melting point agarose on top of 0.7% bottom agarose) in 6-well plates and fed with DMEM. Colonies were counted and photographed after 2 weeks. Cell cycle analyses were performed on propidium iodide stained nuclei using a MoFlo™ XDP-Flow Cytometer (FACS, Beckman Coulter, Inc., Brea, CA). Data were analyzed by single-histogram statistics [31, 48].

### Xenograft

All animal studies were approved by the Animal Care Committee of Wuhan University. Four-week-old female BALB/c nude mice were purchased from SLAC Laboratory Animal Co. (Changsha, China) and maintained in microisolator cages. Tumorigenicity assays and tumor volume measurements were performed as previously described [31]. For xenograft experiments, 10^7^ cells were suspended in 100 μL serum-free DMEM and injected subcutaneously in the flanks of animals (*n* = 5 per group). Tumor growth was monitored every three days for a total period of 30 to 40 days. Tumor volumes were calculated by the equation V (mm^3^) = a × b × c/2, where a is the length, b is the width, and c is the height. Tumors were harvested for DNA, RNA, and protein assays as well as standard pathological studies [27].

### Protein expression analysis

Cell monolayers were trypsinized and washed three times with PBS. Cell pellets were lysed with SDS-PAGE lysis buffer, boiled, and resolved with SDS-PAGE (8–12% polyacrylamide gels). Proteins were then transferred to polyvinylidene fluoride membranes which were blocked with 5% nonfat milk and probed with corresponding antibodies including mouse anti-c-Myc monoclonal antibody (sc-40, 1:500, Santa Cruz Biotechnology, Dallas, TX), mouse anti-c-Myc (phospho S62) monoclonal antibody (ab78318, 1:1,000, Abcam, Cambridge, MA), rabbit anti-MAP3K13 polyclonal antibody (AP8008d, 1:1,000, Abgent, San Diego, CA), mouse anti-A-PARP (Cleaved p25) monoclonal antibody (1051-1, 1:10,000, Epitomics, Burlingame, CA) and mouse anti-GAPDH monoclonal antibody (5632-1, 1:10,000, Epitomics, Burlingame, CA). The blots were then incubated with a 1:5,000 dilution of HRP-conjugated IgG (Santa Cruz Biotechnology Inc.), washed and subjected to chemiluminescent detection as previously described [31, 48].

### TCGA data analysis

Datasets for breast cancer were downloaded from the Cancer Genome Atlas (TCGA) data portal (http://tcga-data.nci.nih.gov) [38–40]. Myc, MAP3K13 and miR-206 expression data were assessed and Kaplan Meier curves were calculated in human breast cancer tissues from the TCGA mRNA or miR-seq data set (*n* = 1044). For MAP3K13, patients within the top 1/3 and bottom 2/3 of the distribution were then assigned as having high and low gene expression, respectively. For Myc, patients with ER- and ER+ were then assigned as having high and low gene expression, respectively. For miR-206, patients with any detectable signal or no signal were then assigned as having positive and negative gene expression, respectively. Significance of individual genes within the high (or positive) and low (or negative) expression groups was calculated using a two-sided *t*-test.

### Statistical analyses

For data analysis, the SPSS statistical package for Windows (SPSS 16), the GraphPad Prism Software (version 5.01) and Microsoft Excel (Excel in Microsoft Office 2013 for Windows) were used. Data are expressed as the mean ± SD or SEM. Other statistical analysis was performed using the two-tailed Student's *t* test, Mann-Whitney *U* test or Log-rank test and *P* < 0.05 was considered statistically significant.

## SUPPLEMENTARY MATERIALS FIGURES AND TABLES


