# Supplementary material for: microRNA-206 impairs c-Myc-driven cancer in a synthetic lethal manner by directly inhibiting MAP3K13
Source: Oncotarget. 2016 Feb 24;7(13):16409–19. doi: 10.18632/oncotarget.7653 (PMC4941324; doi:10.18632/oncotarget.7653)
Supplement: Supplementary file 1 [file oncotarget-07-16409-s001.pdf]

# microRNA-206 impairs c-Myc-driven cancer in a synthetic lethal manner by directly inhibiting MAP3K13

## Supplementary Materials

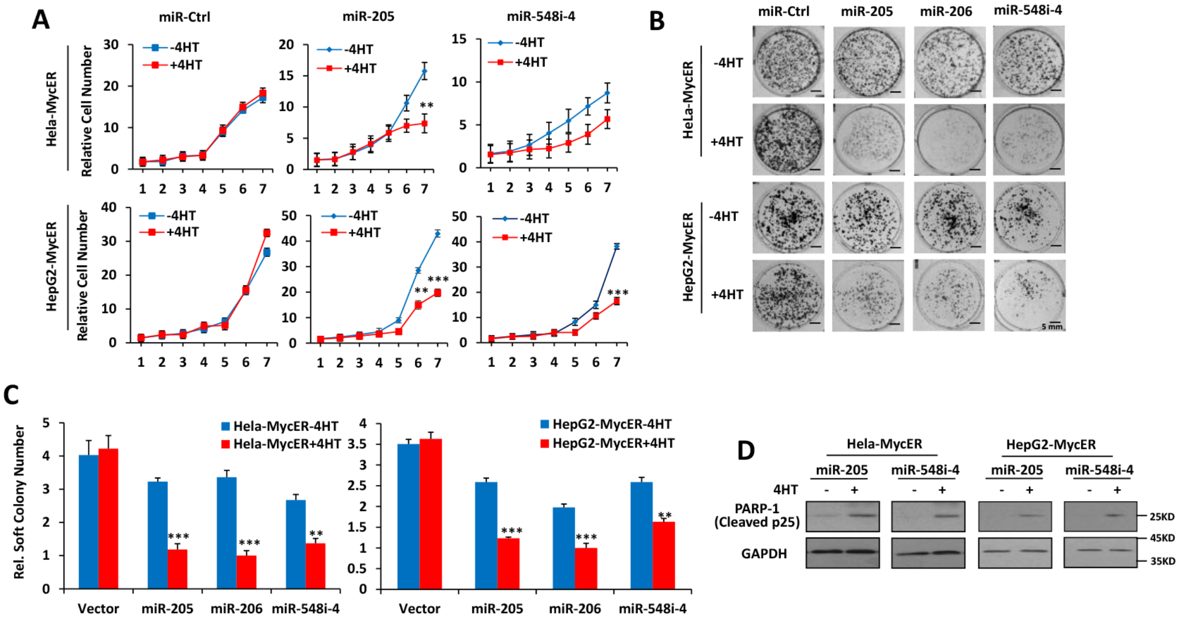


**Supplementary Figure S1 (Related to Figure 2): The effects of miR-205, miR-206 and miR-548i-4 on the growth, anchorage-independent growth and apoptosis in Hela-MycER and HepG2-MycER cells with or without Myc induction.** (**A–C**) Overexpression of miR-205, miR-206 and miR-548i-4 affects growth, foci formation andanchorage-independentsoft agar growth of Myc overexpression cells. (**D**) Overexpression of miR-205, miR-206 and miR-548i-4 promotes apoptosis of Myc overexpression cells. Hela-MycER and HepG2-MycER cells stably transfected with control, miR-205, miR-206 and miR-548i-4 expressing vectors, respectively, were distributed to perform growth curves, foci formation, anchorage-independentsoft agar growth and apoptosis analysis including PARP-1expression detection. Results are means ± SD. $^{*}P < 0.05$, $^{**}P < 0.01$, $^{***}P < 0.001$.

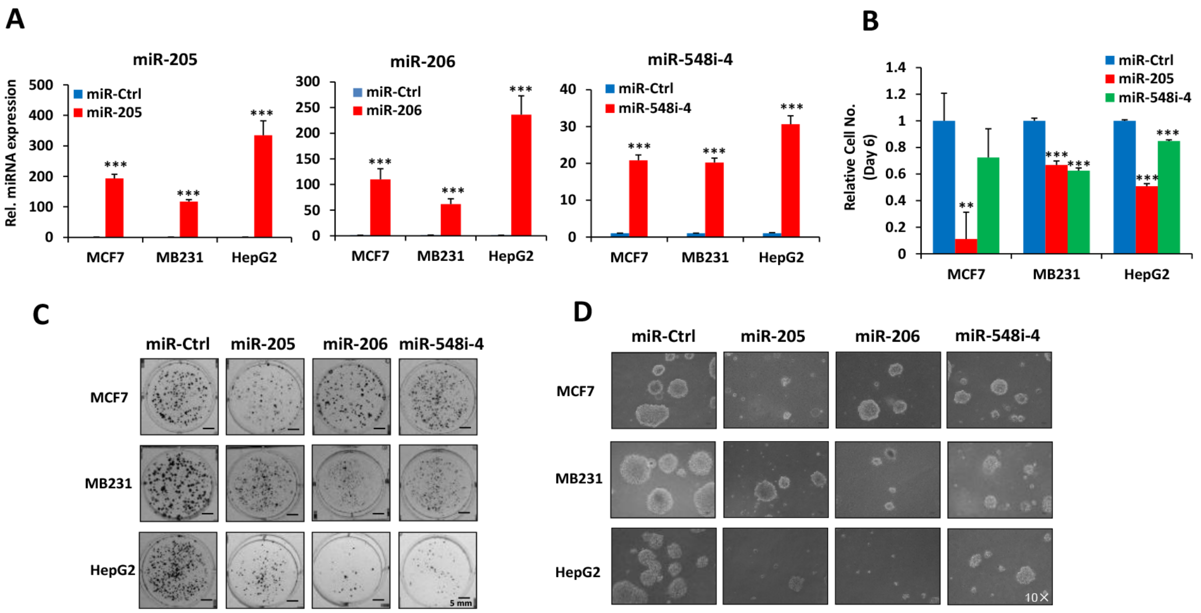


**Supplementary Figure S2 (Related to Figure 3): The effects of miR-205, miR-206 and miR-548i-4 on the growth and anchorage-independent growth of human cancer cells expressing differential level of Myc.** (**A**) The expression of miR-205, miR-206 and miR-548i-4 was determined by qRT-PCR. (**B**–**D**) miR-205, miR-206 and miR-548i-4 affects growth, foci formation and anchorage-independent soft agar growth of human cancer cells expressing high level of Myc. Human breast MCF-7 cancer cells expressing low level of Myc and human breast MB231 and liver HepG2 cancer cells expressing high level of Myc stably expressing miR-205, miR-206, miR-548i-4 and miR-ctrl, respectively, were distributed to perform the experiments of growth curves, foci formation, anchorage-independent soft agar growth and apoptosis detection. Results are means ± SD. *$P < 0.05$, **$P < 0.01$, ***$P < 0.001$.

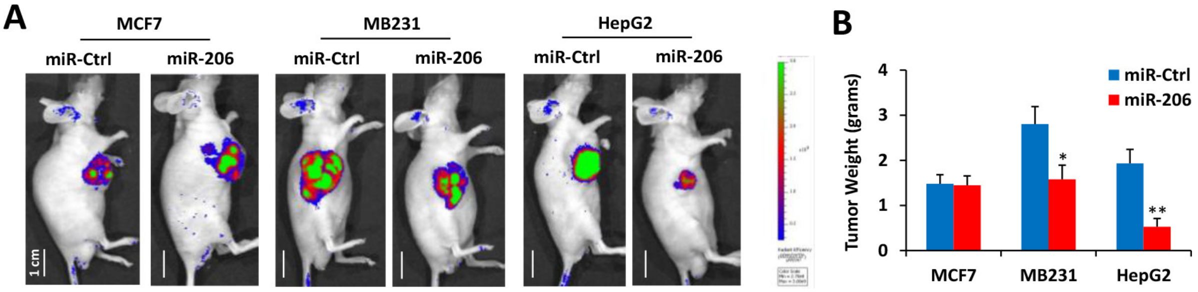


**Supplementary Figure S3 (Related to Figure 3): Overexpression of miR-206 impairs tumorigenicity of human cancer cells expressing high level of Myc.** (**A**) miR-206 inhibited tumor growth of human cancer cells expressing high level of Myc. (**B**) Tumor weight was determined on day 30–40 after sacrificing the mice from the above A. Human breast MCF-7, MB231 and liver HepG2 cancer cells stably expressing miR-206 or miR-ctrl were inoculated into nude mice. Tumor growth was monitered over time. Data are means ± SD. *$P < 0.05$, **$P < 0.01$, ***$P < 0.001$.

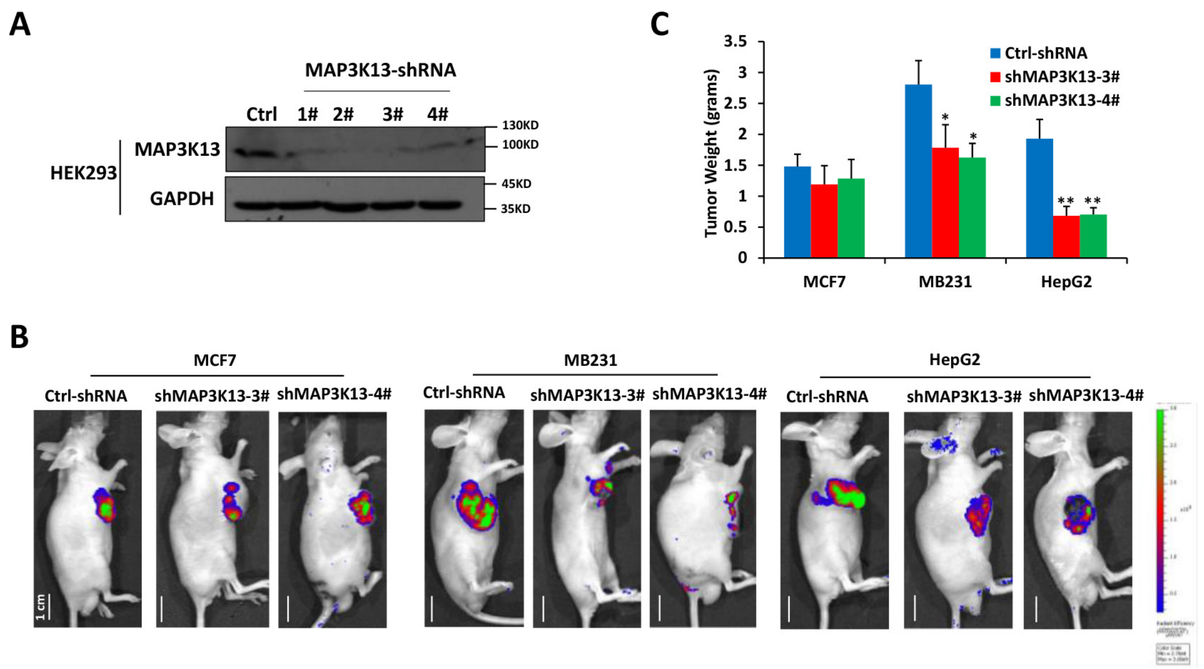


**Supplementary Figure S4 (Related to Figure 5): MAP3K13 is required for tumorigenicity of human cancer cells expressing high level of Myc.** (**A**) Validation of MAP3K13 shRNA.Extracts from HEK293 and HepG2 cancer cells stably expressing differential MAP3K13-shRNA or ctrl-shRNA were assayed with Western blot using MAP3K13 and GAPDH antibodies.MAP3K13-shRNA #3 is the most significant one that inhibited the MAP3K13 expression. MAP3K13-shRNA #3 and #4 was used in the further study. (**B**) Inactivation of MAP3K13 impairs tumorigenicity of human cancer cells expressing high level of Myc.Human breast MCF-7 cancer cells, MB231 and liver HepG2 cancer cells stably expressing MAP3K13-shRNA#3 or ctrl-shRNA were inoculated into nude mice. Tumor growth was monitered over time.(**C**) Tumor weight was detected on day 33–40 after sacrificing the mice from the above (B). Results are means ± SD. *$P < 0.05$, **$P < 0.01$, ***$P < 0.001$.

## Supplementary Table S1 (Related to Figure 1): High content screening results in HeLa-MycER cells using microRNA library

HeLa-MycER cells were seeded in triplicate at a density of 10000 cells per well in 96-well plates for two replicates and transfected with 200 ng individual miRNA expression vector. After 48 hours, cells cultured in the absence or presence of 300 nM 4-OHT. Cell viability was measured 96 hours later using the CellTiter MTS assay. Cells attenuance at 570 nm was read using a plate reader.

## Supplementary Table S2 (Related to Figure 4): Downregulated genes upon enforced expression of miR-206 in HepG2 cells

Total RNA from miR-206 or control miRNA–overexpressing HepG2 cells was amplified and transcribed into fluorescent cRNA. The labeled cRNAs were hybridized onto the Whole Human Genome Oligo Microarray. The arrays were scanned and subsequent data processing was performed to analysis the downregulated genes (2-fold threshold).

## Supplementary Table S3: List of primer and shRNA sequences used in this study

**Primers for construction**

| | Forward Primer | Reverse Primer |
|---|---|---|
| pGL3-MAP3K13-3′UTR-WT | CGCTCTAGACGCACAGGGCTCATG-GATTC | CGCGGATCCAGGCACAGCA CTCTT-GCCCT |
| MAP3K13-3′UTR -MUT | ATTTGATGGACGACTGTAGAAAT | GAATTGTTTATTTCTACAGTCGTC-CATC |
| pHAGE-puro-MAP3K13-Full length | CGCGCGGCCGCATGGC-CAACTTTCAGGAGCACC | CGCGTCGACCCAGGTAGCAGAGCTG-TAGTG |
| pHAGE-puro-MAP3K13-Δkinase domain | CGCGCGGCCGCATGGAAGT-GAAAAAACATTTTGAG | CGCGTCGACCCAGGTAGCAGAGCTG-TAGTG |
| pHAGE-GFP-miR-206 | CGCGGATCCAGTGATCTTCTCGC-TAAGAG | CGCCTCGAGGAAGAAGGTCAC-CAAACCTG |

**qPCR Primers**

| | Forward Primer | Reverse Primer |
|---|---|---|
| MAP3K13 | GAGCTCATTAAGCGTGAGCA | GGGGATCCCTTCTGATCTCA |
| GAPDH | TGCACCACCAACTGCTTAGC | GGCATGGACTGTGGTCATGAG |
| c-Myc | CGTCTCCACACATCAGCACAA | CACTGTCCAACTTGACCCTCTTG |
| GLS1 | GAGACGGACGCGTTTGGCAAC | CAGCAATTGTATAGAACAGC |
| AURKA | GCTGGAGAGCTTAAAATTGCAG | TTTTGTAGGTCTCTTGGTATGTG |
| Cyclin D1 | GCATCTACACCGACAACTC | GGAGAGGAAGTGTTCAATG |

**shRNA sequences**

| ID | Target sequences |
|---|---|
| MAP3K13-shRNA 1# | AGAACAGTATGGGTCCTTA |
| MAP3K13-shRNA 2# | TCCTATCATCCATCCCAAT |
| MAP3K13-shRNA 3# | CCCACAAGAAACTTACTTCAA |
| MAP3K13-shRNA 4# | GCACCCTAACATCATCGCATT |
| Ctrl-shRNA | TTCTCCGAACGTGTCACGT |
